# Shared mechanism of teratogenicity of anti-angiogenic drugs identified in the chicken embryo model

**DOI:** 10.1038/srep30038

**Published:** 2016-07-22

**Authors:** Shaunna L. Beedie, Chris Mahony, Heather M. Walker, Cindy H. Chau, William D. Figg, Neil Vargesson

**Affiliations:** 1 School of Medicine, Medical Sciences and Nutrition, Institute of Medical Sciences, University of Aberdeen, Foresterhill, Aberdeen, UK; 2 Molecular Pharmacology Section, Genitourinary Malignancies Branch, Center for Cancer Research, National Cancer Institute, NIH, Bethesda, MD 20892, USA

## Abstract

Angiogenesis, the formation of new blood vessels, is essential for tumor growth, stabilization and progression. Angiogenesis inhibitors are now widely used in the clinic; however, there are relatively few published studies on the mechanism of their presumed teratogenic effects. To address this issue, we screened a variety of angiogenesis inhibitors in developing zebrafish and chicken embryo models to assess for developmental defects and potential teratogenic effects. We confirmed previous reports that sunitinib, sorafenib and TNP-470 are teratogenic and demonstrate that axitinib, pazopanib, vandetanib, and everolimus are also teratogens in these models. A dose response study identified the drugs inhibit HUVEC cell proliferation *in vitro*, and also target the developing blood vessels of embryos *in vivo*. This provides further evidence for the potential risk of fetal toxicity when using these drugs in a clinical setting, and emphasizes the importance of the development and maintenance of the vasculature in the embryo. We conclude that angiogenesis inhibitors, regardless of the molecular target, are teratogenic when exposed to chicken embryos.

Angiogenesis, the formation of new vessels, is a complex process that has critical importance in physiologic (such as embryogenesis) and pathologic conditions. Several studies have shown a correlation between high levels of angiogenic factors and increased tumor aggression^1^,^2^,^3^. Many current treatments incorporate the use of anti-angiogenic drugs to restrict the vascular supply to the tumor, reducing the mortality and morbidity from cancers by interfering with signaling mechanisms that promote the growth and survival of new blood vessels^4^, and have shown the potential for clinical benefit in a variety of cancers^5^,^6^,^7^,^8^,^9^,^10^. Recent years have seen an increase in the use of these agents for non-cancer conditions where inappropriate angiogenesis is a factor for disease progression; including ocular diseases^11^,^12^, Crohns disease^13^,^14^, as anti-obesity agents^4^,^15^ and have also shown promise in endometriosis^16^,^17^,^18^.

Given that the teratogenic drug thalidomide is thought to produce embryonic defects by inhibition of growth of naive, newly forming blood vessels, it is possible that anti-angiogenic drugs used during pregnancy could induce birth defects in a developing embryo^19^,^20^,^21^,^22^,^23^. Because of this possibility, the regulatory approvals state a contraindication of use during pregnancy within the packaging and safety information. Few data are available on the safety of anti-angiogenic drugs when given during pregnancy including the monoclonal anti-VEGF antibody bevacizumab^24^,^25^. The only available studies in pregnancy come from the use of intravitreal bevacizumab injections in the ophthalmic setting^26^. Bevacizumab has been well-studied in preclinical models which showed teratogenicity in rabbits at twice the recommended intravenous human dose and exhibited embryotoxicity and dose-dependent placental transfer during all gestational ages in rats^27^,^28^. However, there are very limited published reports of other anti-angiogenic targeted agents and their potential teratogenic effects, particularly their mechanism of teratogenicity in preclinical models.

To investigate if anti-angiogenic drugs are teratogenic and to shed light on whether similar or shared mechanisms are used by these drugs to cause teratogenesis, a cohort of targeted agents, currently approved and used to treat patients, or in preclinical development, were screened for teratogenicity using the chicken embryo model system. In addition, we used the stable transgenic zebrafish line, *fli1:EGFP*^29^, where the endothelial cells express green fluorescent protein throughout development, allowing blood vessels to be studied live following drug exposure. Both chicken and zebrafish embryos have been shown to be sensitive to exposure to toxicants and anti-angiogenic compounds during early development^20^,^30^,^31^,^32^,^33^.

We investigated a variety of angiogenesis inhibitors with different molecular targets including the small-molecule tyrosine kinase inhibitors sunitinib, vandetanib, pazopanib, axitinib, and sorafenib, the mammalian target of rapamycin (mTOR) inhibitor everolimus, the methionine aminopeptidase 2 (metAP2) inhibitor TNP-470 (a fumagillin analog) and CPS49 (a thalidomide analog). Previous studies of TNP-470, sunitinib and sorafenib in animal models including mice^34^,^35^, rats, zebrafish and rabbits^36^,^37^,^38^,^39^ and regulatory prescribing information indicates these drugs are potential teratogens (Table 1). Our study aims to determine if all the drugs tested target newly forming vessels to cause defect, and if the defects are comparable, regardless of the differing downstream molecular targets.

We found that every drug showed toxicity and teratogenicity to varying degrees in our developmental models, and inhibited cell proliferation in human umbilical vein endothelial cells (HUVECs) *in vitro*. The most potent compounds were sunitinib, axitinib, pazopanib and sorafenib, which displayed effects at lower concentrations than the other agents. Everolimus, vandetanib and TNP-470 were the least potent in these assays but still induced defects. Mechanistically, all of these compounds were shown to target newly forming blood vessels in the developing embryo, similar to a primary mechanism of thalidomide-induced teratogenesis^20^,^22^. We conclude that these anti-angiogenic compounds have the potential to be teratogenic, exhibit a common mechanism of teratogenicity, which is the loss of correct formation and development of naïve blood vessels, and emphasize that safety in prescribing is crucial for anti-angiogenic drugs, especially in women of childbearing age.

## Results

### Anti-angiogenic compounds decrease endothelial cell proliferation over a concentration gradient *in vitro*

**Figure 1:**
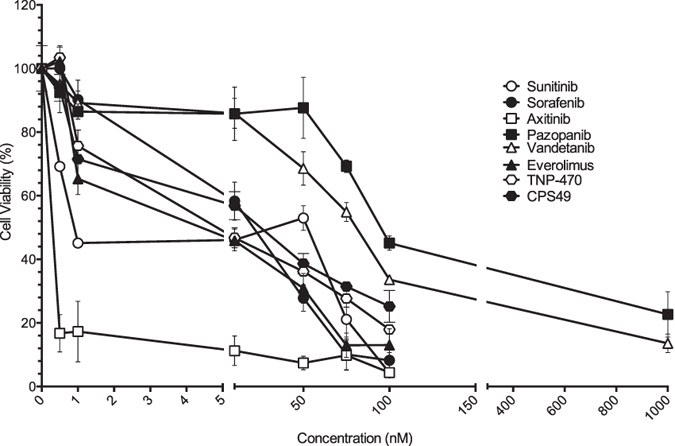
Inhibition of endothelial cell proliferation. Anti-angiogenic drugs were screened in a concentration gradient in the HUVEC culture assay. The drugs maximal activity was within the range 1 nM–1000 nM. Cell proliferation was assessed using a CCK-8 assay (Dojindo). Statistical analysis by two-way Anova. Error represents the standard error of the mean.

In order to confirm the inhibitory effect of the compounds on blood vessel formation and to establish an IC_50_ for the compounds *in vitro* we applied the compounds to an endothelial cell line, Human Umbilical Venous Endothelial Cells (HUVEC) (n = 9). With the exception of vandetanib and pazopanib, all compounds reduced endothelial cell proliferation by 50% at 100 nM or less (Fig. 1). Based on our cell proliferation assay axitinib was seen to be the most potent compound (IC_50_ < 0.5 nM), followed by sunitinib, TNP-470 and everolimus (IC_50_ < 10 nM), and CPS49 and sorafenib (IC_50_ < 50 nM).

### Anti-angiogenic compounds are teratogenic in the developing chicken embryo

**Figure 2:**
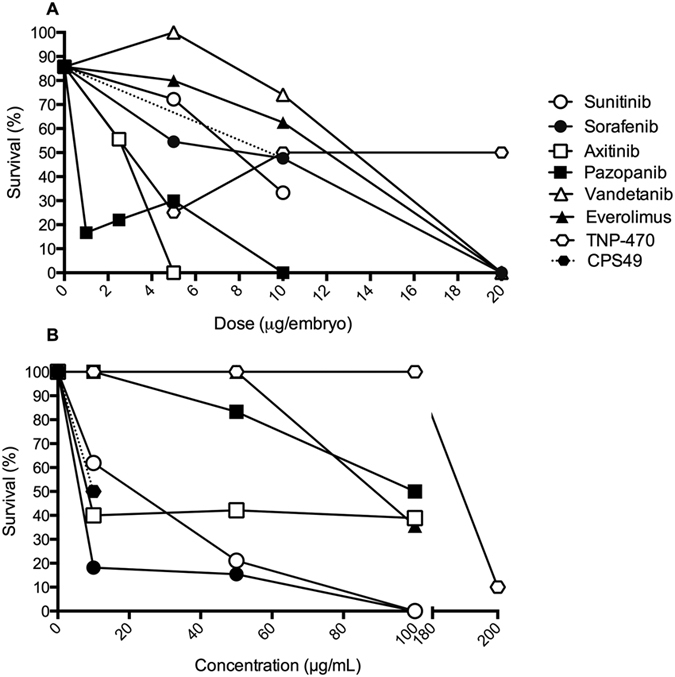
Embryo survival following anti-angiogenic drug exposure. (**A**) Survival of chicken embryos at 48 hours with global treatment over a concentration gradient versus control embryos treated with 0.1% DMSO. (**B**) The survival of *fli1*:EGFP zebrafish embryos at 24 hours after exposure to drug versus control embryos treated with 0.1% DMSO.

We then administered the compounds to developing chicken embryos to observe any teratogenic effects. Chicken embryos have previously been used in drug screening analyses due to their sensitivity to teratogens and their external development allowing easy access to the embryos and rapid monitoring of development^20^,^31^,^32^,^33^,^40^. Each drug was tested over a range of concentrations to establish the dose at which they retained a high survival rate (Fig. 2A) and such concentrations are relative to, or lower than, human therapeutic doses (Table 1). Previous work in our laboratory demonstrated CPS49 has potent anti-angiogenic activity at 10 *μ*g/embryo^20^. Given the highly anti-angiogenic effects of these compounds we initially screened them at the lower concentration of 5 *μ*g/chicken embryo. At 48 hours post drug application both the survival rate and the incidence of defects were analyzed. A range of concentrations was tested to determine the effects and optimal concentration that was not 100% lethal. Embryos treated with just the vehicle 0.1% DMSO showed normal development and high survival rates (85.71%).

**Figure 3:**
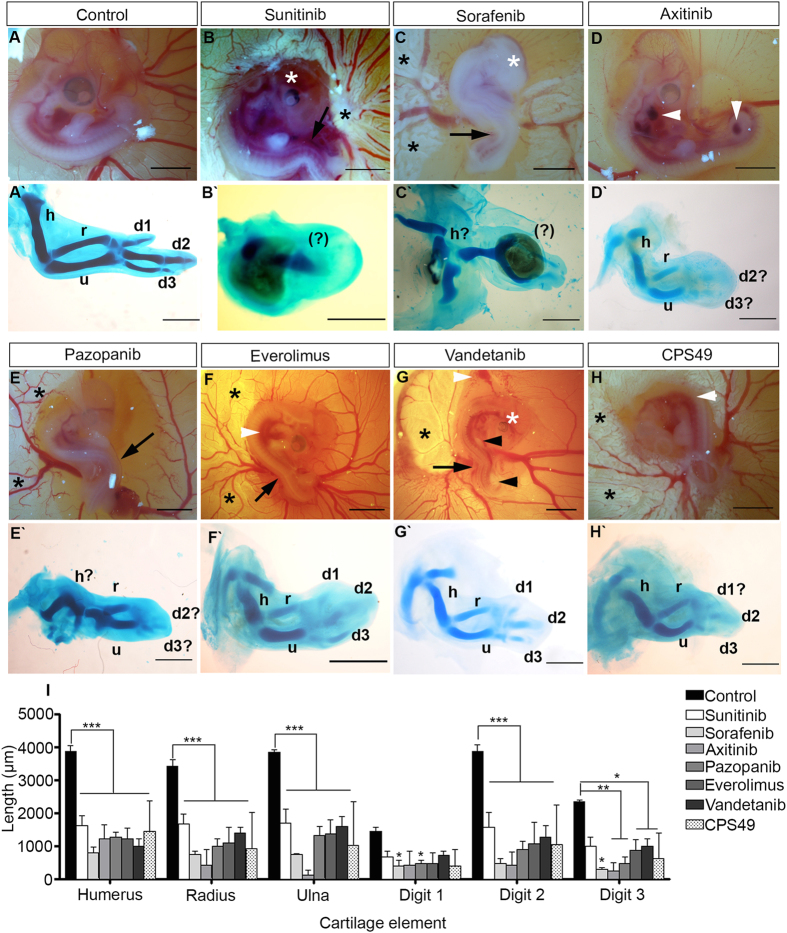
Anti-angiogenic drugs cause tissue damage in chicken embryos. Examples of teratological defects induced with anti-angiogenic compounds. (**A**) Control embryo as imaged *in ovo.* (**A**’) Control cartilage stain; (**B**) Sunitinib treated embryo and resulting (**B**’) cartilage pattern of the limb; (**C**) Sorafenib treated embryo and resulting (**C**’) cartilage pattern of the limb; (**D**) Axitinib treated embryo and resulting (**D**’) cartilage pattern of the limb; (**E**) Pazopanib treated embryo and resulting (**E**’) cartilage pattern of limb; (**F**) Everolimus treated embryo and resulting (**F**’) cartilage pattern of limb; (**G**) Vandetanib treated embryo and resulting (**G**’) cartilage pattern of the limb; (**H**) CPS49 treated embryo and resulting limb cartilage (**H**’) pattern. (**I**) Reductions in cartilage elements of the developing chicken forelimb after treatment with the anti-angiogenic compounds. Labelling: white asterisk indicates eye defect, black asterisk labels necrosis in YSM, white arrow head indicates hemorrhaging, black arrow head indicate limb reduction, black arrow indicates twisting of the spinal cord; cartilage patterns: (h) humerus, (r) radius, (u) ulna, (d1-3) digits 1, 2 and 3, and (?) represents an unknown cartilage element. Scale bars: **A,B,D–H**: 1000 μm, **C**: 500 μm, (**A**’–**H**’) 500 μm. Error represents the standard error of the mean where ns = p > 0.05, *p < 0.05, **p < 0.01, ***p < 0.001.

In contrast, when treated with sunitinib (5 *μ*g/embryo) the survival rate was reduced to 72%. Of those surviving, in most embryos limb defects were observed, some also exhibited microopthalmia and spinal cord twisting (Fig. 3B). We also observed necrotic or dead areas in the surrounding yolk sac membrane (YSM) (Fig. 3) which have also been recorded in other studies of anti-angiogenic drugs^20^. Similar results were seen with sorafenib application (Fig. 3C), at 5 *μ*g/embryo the survival rate decreased to 54.5% and at 10 *μ*g/embryo survival was reduced to 47%. Following axitinib application at 5 *μ*g/embryo, no embryos survived the treatment. At 2.5 *μ*g/embryo the survival rate increased to 55.5%. Defects were again seen, in particular most embryos exhibited necrosis within the surrounding YSM and hemorrhaging (Fig. 3D). Pazopanib treatment at 5 *μ*g/embryo led to a low survival rate (30%) so the compound was then administered at the lower concentrations of 2.5 *μ*g/embryo, where 22% of the treated embryos survived and then at 1 *μ*g/embryo, where the survival rate was 16%. Embryos treated with this drug had malformation of the blood vessels and necrosis in the YSM, and twisted spinal cords (Fig. 3E). Everolimus showed no impact on the survival rate at 5 *μ*g/embryo with an 80% survival rate but at 10 *μ*g/embryo survival decreased to 62.5%. Embryos treated with everolimus also had hemorrhaging, spinal cord twisting and necrosis in the YSM (Fig. 3F). Whilst having the highest survival rate of all treatment groups (no death occurred at 5 *μ*g/embryo) vandetanib still induced defects in treated embryos, such as limb reductions, micropthalmia, twisted spinal cords, and necrosis and hemorrhaging in the YSM, and was shown to decrease the survival rate at 10 *μ*g/embryo (74% survival). TNP-470 at 5 *μ*g/embryo had a survival rate of 25%. At 10 *μ*g/embryo reduced survival to 50% and caused twisting of the spinal cord. At the higher concentrations of 20 *μ*g/embryo survival was again 50%.

Our results showed that all the drugs tested in this study are detrimental to the development of chicken embryos. Additionally at high concentrations every drug screened is lethal to the developing embryo, at the developmental time point tested. In this assay, all compounds caused defects, and all defects were comparable to those induced by CPS49 (Fig. 3H).

### Anti-angiogenic compounds cause limb defects when applied directly to the developing limb bud of chicken embryos

Given the potency of these drugs and the resulting embryonic lethality we focused our attention on how the drugs affect limb development. The developing limb bud is a well studied morphological assay^22^,^41^,^42^ ideal for understanding mechanisms of embryogenesis and how drugs influence this process. Moreover, other anti-angiogenic drugs like thalidomide and valproate cause limb defects^21^,^42^,^43^,^44^. Here we wanted to investigate if the test compounds cause limb defects and if the damage they induce are comparable to defects caused by other anti-angiogenic drugs. We therefore established a method where a small filter paper square soaked in the drug of interest could be placed directly onto the limb tissue of the embryo, thus avoiding or minimalizing exposure of the drug to other developing tissue/structures and retaining high viability. Filter paper squares were soaked for 10 minutes prior to removal of the embryonic membrane of HH St 18–20 (E2.5-E3) chicken embryos, then placed over the developing limb bud. Filter paper squares soaked in 0.1% DMSO had no effect on limb outgrowth. Tissue was necrotic-looking in TNP-470 treated embryos, and as such we were not able to ascertain the cartilage patterns in these embryos.

Utilizing this methodology we found that when the developing limb buds of chicken embryos are exposed directly to the test compounds they disrupted the patterning of the limb, leading to loss of cartilage and developmental malformations (Fig. 3A’–H’). In cases where the filter paper sponge was placed close to the YSM, malformation of the vessels near the drug soaked sponge could be observed, similar to destruction of the YSM seen in global treatment, confirming the potent anti-angiogenic effects.

These *in vivo* chicken embryology experiments demonstrate that all of the anti-angiogenic drugs examined were teratogenic to the developing chicken embryo. When the limbs of chicken embryos are exposed to these drugs they induce limb defects, which can be reminiscent to those formed by the human teratogen thalidomide, as seen by loss and reduction of limb elements^20^,^22^,^40^,^42^.

### Anti-angiogenic compounds target the developing vasculature of zebrafish embryos

**Figure 4:**
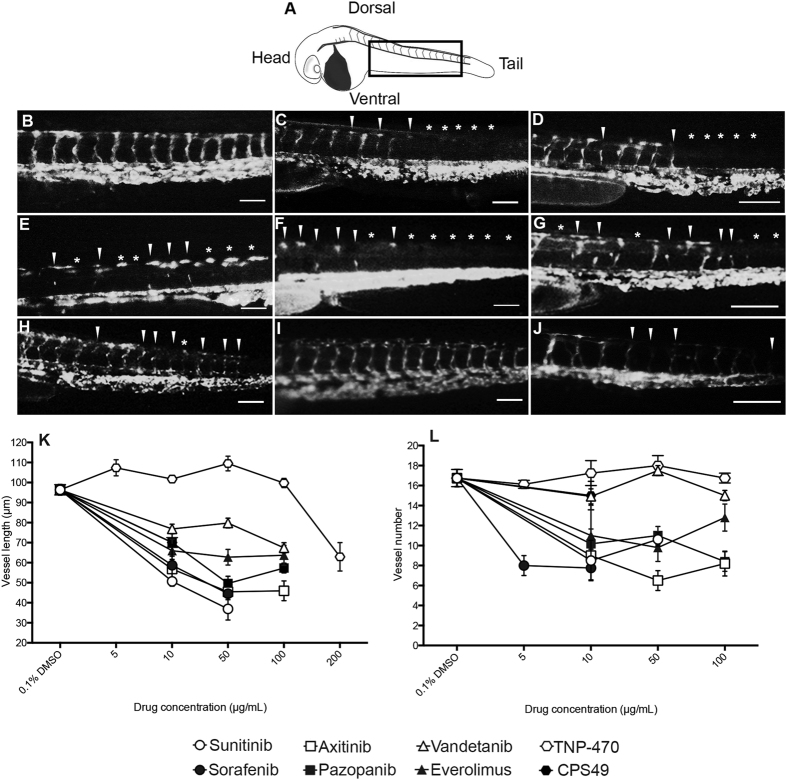
Anti-angiogenic anti-cancer drugs target intersomitic vessel outgrowth in fli1:EGFP embryos. Schematic of zebrafish embryo at 48 hpf. Black rectangular box indicates the imaging area used within this figure. (**B**) Treatment with 0.1% DMSO, (**C**) vandetanib (100 μg/mL), (**D**) sunitinib (100 μg/mL), (**E**) sorafenib (100 μg/mL), (**F**) axitinib (100 μg/mL), (**G**) pazopanib (100 μg/mL), (**H**) everolimus (100 μg/mL), (**I**) TNP-470 (100 μg/mL), and (**J**) CPS49 (10 μg/mL). (**K**) Vessel outgrowth and (**L**) number is reduced with anti-angiogenic treatment over a concentration gradient, with the exception of TNP-470 and vandetanib for vessel number. White arrow-heads show reduction in vessel outgrowth, white asterisks show complete loss of vessel. Scale bar = 100 μm.

We then examined the extent and nature of the anti-angiogenic effects of the compounds using transgenic *fli1*:EGFP zebrafish embryos. The vasculature of these fish is tagged with EGFP and the transparency of the zebrafish allows for visualization of the forming blood vessels and any effect of anti-cancer drugs on their development over time (Fig. 4). A known anti-angiogenic and teratogenic compound (CPS49) was screened alongside the drugs as a positive control at 10 μg/mL (30 μM), a previously established anti-angiogenic concentration^20^. Embryos treated with CPS49 had a significant reduction in both blood vessel outgrowth and the number of forming vessels (Fig. 4J) when compared to vehicle control (0.1% DMSO) treated embryos (Fig. 4B). The embryonic survival rate also decreased to 50% (Fig. 2B).

Treatment with all of the compounds blocked vasculature development, as characterized by a decrease in both stalk number and outgrowth (Fig. 4B–L). Like CPS49, the survival rates of treated embryos were also reduced compared to control embryos (Fig. 2B). When treated with sunitinib (Fig. 4D) at 10, 50 and 100 μg/mL, survival decreased to 61.90%, 21.05%, and 0% respectively. With sorafenib (Fig. 4E), at 10 and 50 μg/mL the survival dropped to 18.18% and 15.38%. No embryos survived treatment at 100 μg/mL. Treatment with axitinib (Fig. 4F) decreased the survival to similar numbers at all concentrations tested (10 μg/mL, 40%; 50 μg/mL, 42.12%; 100 μg/mL, 38.89%). Vandetanib (Fig. 4C) treatment at 10 μg/mL reduced survival to 60%, at 50 μg/mL it was reduced to 15.38% and at 100 μg/mL it dropped again to 10%. Treatment with everolimus (Fig. 4H) did not impact the survival of the embryos at 10 μg/mL or 50 μg/mL however at 100 μg/mL survival was reduced to 35.71%. Pazopanib (Fig. 4G) also did not affect the survival at 10 μg/mL, but did at 50 μg/mL (83.33%) and 100 μg/mL (50%). At low doses (<200 μg/mL) TNP-470 (Fig. 4I) was shown to have little effect. At 200 μg/mL the drug became highly toxic, killing 90% of treated zebrafish, and reducing vessel outgrowth.

These data demonstrate that these compounds affect embryonic blood vessels rapidly within 24 hours of drug exposure. The drugs appear to be more anti-angiogenic than CPS49 on *fli1*:EGFP embryos. Of the compounds screened sunitinib, sorafenib, axitinib and pazopanib were the most potent, reducing vessel outgrowth at similar concentrations (Fig. 4K). Vandetanib and everolimus were the least potent- with the exception of TNP-470, which had no effect until 200 μg/mL, when it was seen to be toxic, as indicated by high lethality in zebrafish (Fig. 2B).

## Discussion

Angiogenesis inhibitors are finding widespread use in the clinic; given the paucity of published preclinical studies evaluating the mechanism of their presumed teratogenic effects we screened several targeted agents in the developing chicken and zebrafish embryo models to assess the developmental defects. We identify differences in potency between them as well as show they are potentially teratogenic. Utilizing *in vitro* cell culture experiments we confirmed the anti-proliferative activities of the compounds in endothelial cells and compared them to the anti-angiogenic thalidomide analog CPS49. We demonstrated *in vivo* in the developing *fli1*:EGFP zebrafish embryo that these drugs target the newly developing vasculature of the embryos over a range of concentrations. We showed that the compounds were teratogenic to the developing chicken embryo and in some cases embryolethal to zebrafish embryos.

Based on our results we observe that the anti-angiogenic potential of each inhibitor may determine the extent of defects produced in the animal models. For example, axitinib and sunitinib were the most anti-proliferative in endothelial cells, and caused more endothelial cell death than vandetanib, which was not as anti-proliferative at the same concentrations. However, this *in vitro* assay was only somewhat predictive, particularly in the case of pazopanib, which had one of the highest IC50s *in vitro* but was highly anti-angiogenic *in vivo*, embryotoxic and teratogenic. Combining the *in vitro* data with the vessel development in the transgenic zebrafish gave a better indication of teratogenic potential. For example, sunitinib showed the best response in the *fli1*:EGFP zebrafish model, vastly reducing outgrowth of naïve, newly sprouting blood vessels, and consequentially caused malformations in the developing chicken embryo associated with misregulation of vasculature developing including limb, spinal, microphthalmia, growth inhibition, necrosis of the YSM (Table 1).

When applied globally to chicken embryos the drugs induced tissue damage to other areas of the embryo, in particular twisting in the spinal cord and causing developmental delay (Table 1). At high concentrations the drugs were embryolethal, indicating the toxicity of these drugs (Fig. 4), and may indicate the possibility of these drugs to cause miscarriage. We also show than when exposed directly to the developing limb bud these drugs have the ability to induce limb reduction damage, which share similarities to those induced by thalidomide and some analogs in previous chicken embryo studies^20^,^39^. For TNP-470, at all concentrations tested the treated embryos could not survive the treatment long enough to allow for analysis of cartilage patterns, indicating the potency and embryolethality of TNP-470 in this model. Mouse models examining TNP-470 during pregnancy have shown the compound to be anti-angiogenic, disrupting newly developing blood vessels in the placenta and embryonic eye^34^. The differences in potency of this compound may be due to species specificity, or the method of application. We hypothesized that the cause of death may be due to the drugs causing a fatal hemorrhage in one or more of the major blood vessels within the embryo, but further studies would be required to establish the specific cause.

Nonetheless, our study demonstrates that anti-angiogenic drugs induced defects in the developing chicken embryos. Other anti-angiogenic agents also cause similar damage to embryos, including, exposure to thalidomide and some thalidomide analogs^21^,^22^,^32^,^33^,^40^, CPS49^20^ as well as agents such as sodium valproate^44^. This data suggests that angiogenesis inhibitors, regardless of the molecular target, exhibit a common mechanism of teratogenicity- the loss of newly formed or forming blood vessels similar to thalidomide-induced teratogenesis with the developing limbs being particularly susceptible because of their relatively immature, highly angiogenic vessel network^20^,^41^,^42^.

While the production of defects are dependent on the drug applied and may indicate a variety of molecular targets or differences in action for teratogenic drugs, it has previously been shown *in vitro* that five of the compounds tested in this study act through manipulation of VEGF receptor signaling, either at the receptor itself or on a downstream target^45^. As thalidomide and its analogs could also influence VEGF receptor signaling via modulation of COX2^46^, this data may imply a common downstream pathway for both anti-angiogenic and teratogenic effects. Interestingly, everolimus does not act through the VEGF receptor, but rather as an inhibitor of mTOR, a downstream molecule of the VEGFR signaling pathway. Of the compounds screened everolimus was seen to be one of the least teratogenic, suggesting that specific targeting of downstream target molecules may be less detrimental to the developing vasculature than a general VEGF receptor (or multiple kinase receptor) inhibitor.

Our study highlights the effectiveness and benefits of the developing chicken and zebrafish embryos as model organisms for screening of developmental toxicity and embryotoxic potency of compounds during early drug development. The relative ease with which the embryos can be obtained and maintained, as well as the low cost of upkeep, and the rapid time to development allow for quick screening of a large amount of compounds. The uniformity of development of these species also allows for the comparison of drugs, and these models have been previously used to distinguish teratogenic profiles of different classes of compounds. We found that when exposed to the test compounds the chicken embryos were affected more severely than the zebrafish embryos. A possible explanation for this is the method of application. A solution of each drug was applied over the body of the chicken embryo, whereas the drugs were diluted in the water of the zebrafish embryos. While some drug may wash off from the chicken embryos, it is possible they receive more of the compound than the zebrafish due to the direct application of the drugs. It should also be noted that some drugs (including thalidomide) are known to have species-specific differences in action. Their may be species-specific effects regarding the toxicity of byproducts and structural analogs of the drug, for example, pomalidomide at potent anti-inflammatory doses, is not teratogenic in chicken embryos, yet is in some mammalian species^31^.

The systems we use here accurately model the data from animal studies reported in the regulatory drug package inserts (Table 1). It should be noted that the concentrations tested here are not comparable to the concentrations tested in animals. Concentrations applied were lower (rat treatment is approximately 40 mg/kg) but the defects produced by compounds in the developing chicken embryo assay correlated to those produced in rodent and rabbit studies (including high death rate, skeletal malformations, microphthalmia). The ability of chicken embryos to produce embryonic defects such as those seen when tested in higher animal species emphasizes the practical uses of our models for preliminary identification of potential teratogens, but thorough testing in higher species remains essential.

Many countries have programs to limit the chance of exposure to teratogens (for example, the S.T.E.P.S program utilized in the U.S. for the safe prescribing of thalidomide) but most anti-angiogenic drugs are not used with such programs. Moreover, there is an increased risk of an epidemic of children being born with defects in countries where there are reduced restrictions on drug availability, and where drug sharing is common practice (such as Brazil)^21^,^22^,^47^,^48^,^49^. The increased incidence of anti-angiogenic drug use requires safeguards in prescribing^23^,^50^. Additionally, for the majority of drugs approved for use in the U.S., the teratogenic risks in human pregnancy are unknown^51^; thus, thorough drug screening to identify potential teratogenic drugs and/or common mechanisms of teratogenicity is warranted.

In conclusion, we have demonstrated that different classes of anti-angiogenic drugs are teratogenic and cause surprisingly similar damage in chicken embryos, suggesting similar or shared mechanisms resulting in teratogenesis. These results provide support for a concept and screening process using the developing chicken embryo and zebrafish embryo as validated model systems to screen novel angiogenesis inhibitors undergoing preclinical development in order to identify a potential teratogenic profile and to promote the safety of a diverse spectrum of drugs with anti-angiogenic activity in women of childbearing age.

## Materials and Methods

### Materials

Drugs were purchased from LC Laboratories (Woburn, MA). When required, the drugs were dissolved in dimethyl sulfoxide (DMSO) to 40–100 mg/mL. When applied to embryos, the drug was further dissolved in sterile PBS giving a maximum final concentration of 0.1% DMSO (previously shown to be non toxic^20^. The known anti-angiogenic thalidomide analog CPS49 was used as a positive control, as it has been previously shown to induce developmental defects in both assays and show anti-angiogenic activity *in vitro*^20^,^52^,^53^.

### Endothelial Cell Proliferation Assay

HUVEC cells were purchased from ATCC (Manassas, VA) and cultured as previously described^30^. No further authentication was performed. Cells were seeded at 6.5 × 10^4^ cells per well in 100 μL of medium per well. Drugs were diluted in prewarmed DMEM. Serial dilutions were performed from 1000–0.1 nM. The cells were left to grow for 18 hours with exposure to vehicle control (DMSO) or drugs. Experiments were done in triplicate each time. Cellular proliferation was assessed using a CCK-8 assay (Dojindo, Rockville, MD). All plates were read at 450 nm.

### Chicken embryology experiments

Fertilized white leghorn chicken embryos purchased from Henry Stewart Ltd. (Herefordshire, United Kingdom) were maintained in an incubator as described previously^31^. At Hamburger and Hamilton stage 18–20 (E2.5-3)^54^ the eggs were windowed and the embryos were prepared for drug exposure. To establish a dose response the drugs were applied once to the embryos and growth was monitored as described previously^31^. In a second set of experiments, filter paper squares were cut 100 μm × 100 μm and soaked in the compound of interest for 10 minutes. While the filter paper squares absorbed the solution, embryonic membranes were removed and the sponge was then placed onto the limb bud. The eggs were sealed with tape and returned to an incubator. The embryos were checked daily for developmental defects and to ensure the filter paper squares remained in place. Control embryos were treated with 100 μL PBS with 0.1% DMSO. Embryos were removed from the eggs and fixed in 5% TCA at 4 degrees for 24 hours. Embryos were then washed in alcian blue for 8 hours, and placed in acid ethanol over night before being dehydrated in 100% ethanol for three one hour treatments. The tissue was then cleared in methyl salicylate. Cartilage was imaged and subsequently measured using a graticule scale and ImageJ. A minimum of 8 embryos was used per drug, per concentration. All animal work was licensed and carried out in accordance with and with approval of UK Home Office and Institutional ethical and welfare guidelines.

### Zebrafish embryology

*fli1*:EGFP zebrafish were obtained from the Zebrafish international Resource Centre^29^ and maintained in an approved aquarium habitat at 28 °C. At 24 hours post fertilization (hpf) the embryos were checked for GFP expression using a fluorescent microscope and manually dechorionated with forceps, exposed to either a vehicle control, or an anti-angiogenic anti-cancer compound (at 24 hpf) for 24 hours and imaged at 48 hpf. At this point the embryos were anaesthetized in 0.1% tricaine and the length and number of intersegmental vessels (ISV) were quantified as previously described^31^. A minimum of 10 embryos were screened per drug, per concentration, and experiments were performed in triplicate. All animal work was licensed and carried out in accordance with and with approval of UK Home Office and Institutional ethical and welfare guidelines.

### Imaging and analysis

Photography was performed used a Nikon SMZ1500 fluorescent microscope. All measurements were completed using Image J software and analyses conducted through Graphpad Prism. For statistical considerations all results were presented as mean with standard error of the mean (SEM), and p < 0.05 as the criterion for statistical significance. Figures were prepared using Adobe Photoshop.

## Additional Information

**How to cite this article**: Beedie, S. L. *et al*. Shared mechanism of teratogenicity of anti-angiogenic drugs identified in the chicken embryo model. *Sci. Rep.* **6**, 30038; doi: 10.1038/srep30038 (2016).

## Figures and Tables

**Table 1:** A comparison of toxicities and defects produced by each anti-angiogenic and anti-cancer drug.

Anti-angiogenic drug^(REF)^	Primary target/s of action	Approved Indications	Clinical Toxicities	Defects in animal models			Defects produced in chicken embryos
				Animal^(REF)^	Systemic exposure (AUC) compared to human exposure	Defects produced	
Sunitinib^45^	Inhibition of VEGFR1/2/3, PDGFRα/β	Pancreatic neuroendocrine tumors, kidney cancer, gastrointestinal stromal tumor	Cardiotoxicity	Rat^55^	5.5×	Embryolethality increased, fetal skeletal malformations (ribs/vertebrae)	Embryolethality increased, limb, spinal, microopthalmia, growth inhibition, necrosis of YSM
				Rabbit^55^	0.3×	Increased embryolethality, cleft palate malformations	
Sorafenib^45^	Inhibition of CRAF, BRAF, VEGFR1/2/3, PDGFRβ	Thyroid cancer, liver cancer, kidney cancer	Cardiotoxicity	Rat^56^	0.008×	Increased post implantation loss, resorptions, skeletal retardations, retarded fetal weight	Embryolethality increased, limb, growth inhibition, necrosis of YSM
				Rabbit^56^			
Axitinib^57^	VEGFR1/2/3	Renal cell carcinoma	Cardiotoxicity	Mouse^58^	10×	Increased post implantation loss, cleft palate malformation, variation in skeletal ossification	Embryolethality increased, limb, growth inhibition, necrosis of YSM
Pazopanib^59^	VEGFR1/2/3, PDGFR	Advanced soft tissue sarcoma, advanced renal cell carcinoma	Cardiotoxicity	Rat^60^	0.1×	Teratogenic, embryotoxic, fetotoxic & abortifacient. Cardiovascular malformations, incomplete/absent ossification, reduced fetal body weight, pre- and post-implantation embryolethality	Embryolethality increased, limb, spine, necrosis of YSM
				Rabbit^60^	0.02×	Increased post-implantation loss, abortion, 100% litter loss	
Vandetanib^61^	VEGFR2, EGFR	Unresectable, locally advanced or metastatic medullary thyroid cancer	Cardiotoxicity	Rat^62^	0.03×−0.4×	Increased pre- & post-implantation loss, malformations of heart vessels, skeletal variations, delayed ossification of the skull, vertebrae & sternum.	Embryolethality increased, limb, spine, microopthalmia, necrosis of YSM
Everolimus^63^	Mammalian target of rapamycin (mTOR)	Subependymal giant cell astrocytoma, advanced hormone receptor positive/HER2-negative breast cancer, progressive neuroendocrine tumors of pancreatic origin, advanced renal cell carcinoma	Stomatitis, noninfectious pneumonitis, rash, hyperglycemia and immunosuppression	Rat^64^	0.04×	Increased resorption, pre- and post-implantation loss, decreased numbers of live fetuses, malformation and retarded skeletal development	Embryolethality increased, limb, spine, microopthalmia, necrosis of YSM
				Rabbit^64^	1.6×	Increased resorptions	
TNP-470^65^	MetAP2	Not approved	Neurotoxicity	Mice^65^	Below human	Complete failure of embryonic growth	Embryolethality, cranial hemorrhaging, micropthalmia, limb defects, spine
Thalidomide^22^,^66^/CPS49^42^,^67^	Cereblon, Tubulin, sGC^22^/unknown	Approved- multiple myeloma/Not approved	Somnolence, constipation, neuropathy, venous thromboembolism and rash/unknown	Mouse^66^ (strain specific) Rabbit/Chicken^20^,^22^	At recommended human dose/Not applicable	Increased resorptions, embryotoxicity, eye defects	Embryolethality, cranial hemorrhaging, micropthalmia, limb defects, spine

A brief summary of the main mechanism(s) of action of each drug screened and their clinical uses are given. Also shown are human toxicities, the defects produced when tested in pregnant animals, including rodents and rabbits, and defects produced in the developing chicken embryo model.
